# Contested futures: envisioning “Personalized,” “Stratified,” and “Precision” medicine

**DOI:** 10.1080/14636778.2019.1637720

**Published:** 2019-07-12

**Authors:** Sonja Erikainen, Sarah Chan

**Affiliations:** Usher Institute of Population Health Sciences and Informatics, College of Medicine and Veterinary Medicine, University of Edinburgh, Edinburgh, UK

**Keywords:** personalized, stratified, precision, expectations

## Abstract

In recent years, discourses around “personalized,” “stratified,” and “precision” medicine have proliferated. These concepts broadly refer to the translational potential carried by new data-intensive biomedical research modes. Each describes expectations about the future of medicine and healthcare that data-intensive innovation promises to bring forth. The definitions and uses of the concepts are, however, plural, contested and characterized by diverse ideas about the kinds of futures that are desired and desirable. In this paper, we unpack key disputes around the “personalized,” “stratified,” and “precision” terms, and map the epistemic, political and economic contexts that structure them as well as the different roles attributed to patients and citizens in competing future imaginaries. We show the ethical and value baggage embedded within the promises that are manufactured through terminological choices and argue that the context and future-oriented nature of these choices helps to understanding how data-intensive biomedical innovations are made socially meaningful.

The last few decades have seen a proliferation of academic publications and policy discourses around so-called “personalized,” “stratified,” or “precision” medicine. While definitions of these terms are often debated, they all broadly refer to the possibilities that are opened up by the translation of emerging modes of data-intensive biomedical research to the context of medical practice and healthcare delivery. This is contextualized by the so-called “digital era” of bioinformatics and “big data” which is enabling the collation of, and application of predictive modeling methods to, unprecedented quantities of biomedical data. The rise of these technologies is associated with hopes and aspirations of a revolutionary change in medicine that will enable new, transformed modes of healthcare delivery in the future. “Personalized,” “stratified,” and “precision” medicine each describe, in different ways, the kinds of future medicine and healthcare that these new methods promise to bring forth.

The concepts of “personalized,” “stratified,” and “precision” medicine and the discourses associated with each are overlapping and future-oriented, characterized by diverse visions of possible, desired and desirable futures that are illustrated by the plurality of terms. These visions in turn are articulated in different ways in different political and epistemic contexts, by different actors with different interests and agendas. The meanings of “personalization,” “stratification,” and “precision” are emergent, without collectively agreed boundaries: they refer to overlapping phenomena and collate shared themes, but each has a different emphasis, which delineates what is and is not included under each term in practice. Each reflects different views of what the aims of emerging biomedical research modes are and should be in the context of healthcare, and positions patients and citizens differently with respect to health and biomedicine as well as in relation to governance and the state.

Building on and contributing to existing literature on the sociology of expectations, our objectives in this paper are, firstly, to delineate key aspects of the negotiations and disputes attached to “personalized,” “stratified,” and “precision” medicine, in order to analyze the divergent and overlapping themes that structure the related discourses. We aim to unpack the various aspirations and epistemic agendas attached to them, and to show how and why different terms are mobilized in policy discourses as opposed to biomedical academic discourses. Second, we map the political, and economic contexts that structure different policy articulations of “personalized,” “stratified,” and “precision” medicine along the lines of US vs Europe differences and similarities, as the articulations have largely originated from and are advanced by US and European political actors. We show how politico-economic interests and cultural differences between the US and European policy contexts frame how different future promises are imagined. Third, we aim to show how the patients and citizen subjects that are the target of medical interventions and healthcare systems are configured differently within and through the competing discourses and imaginaries.

By providing insight into how (the concepts of) “personalized,” “stratified,” and “precision” medicine are delineated and mobilized, we examine how the future-oriented content of the discourses is likely to shape the direction of biomedical innovation today. What aspirations do different politically, epistemically, and geographically positioned actors attach to new and emerging biomedical research modes? What role do these aspirations play in envisioning the future of medicine and healthcare? How are different visions of this future embedded within and shaped by broader contexts in which they are articulated? How are individuals and citizens being (re)positioned with respect to health, healthcare systems and biomedicine? Through charting this contested conceptual landscape, we explore how the dispute over labels and definitions reflects political, social, epistemological and ontological tensions that must be negotiated as part of making sense of techno-scientific and biomedical innovation. We argue that analysis of such innovation and its implications must account, not just for this diversity of terms and the contextual differences that underlie it, but also for the ethical and value “baggage” embedded with the future promises that are manufactured through terminological choices.

## Big data, systems biomedicine, and the sociology of expectations

The emergence of data-intensive biomedical methodologies and predictive analytics is contextualized by the “digital era” of scientific and technological innovation that has exponentially increased computational processing power and enabled the development of complex digital and interactive communication systems and high-throughput technologies including genome sequencing and -omics disciplines. Digital data collection, collation and analysis tools have enabled new research modes that rely on “big data,” i.e. large quantities of varied, dynamic, and mobilizable data (Kitchin 2014). New possibilities for constructing and collating datasets on an unprecedented scope and scale enable the integration of myriad types of data ranging from electronic medical records to individual and population genomics data to environmental sensor and lifestyle data from personal mobile devises and applications (Lupton 2013). Some have argued that big data even facilitates so-called “hypothesis free” research, based on patters and correlations emerging from the data rather than on hypothesized causal pathways (e.g. Baxter, Krokosky, and Terry 2011). Many emerging research methods for making sense of big data have focused on the development of analysis tools and algorithms that enable the integration of diverse data types to construct predictive models of health and disease. While often based on the collation and combined analysis of population data, this kind of predictive modeling tends to be aimed at translating big health data into more targeted and focused – or personalized, stratified, or precise – medical and healthcare interventions for individual subjects or groups (Flores *et al*. 2013).

These new technologies and methods have altered the focus of much biomedical research, giving rise to an epistemic and ontological emphasis on “systems” or networks. This is rooted in the emergence of so-called systems biology, focused on the dynamics of complex biological systems rather than individual parts or constituents of the system. The application of systems approaches to medical and clinical contexts has been termed “systems medicine.” Both entail an ontological and epistemological re-direction in biomedicine (see Fujimura 2005; Green 2017; Powell *et al*. 2007). They aim to move away from “object centered” approaches that place emphasis on ontology, such as “molecular” or “cell” biology where the definition focuses on the object of study, or medical specializations around an object of investigation, such as cardiology or oncology. Rather, emphasis is directed at “systems” as a methodological and epistemological approach and on the construction of models of biological and disease pathways, which can enable a predictive rather than responsive approach to medicine. These emerging research modes are interdisciplinary, requiring collaboration between researchers with diverse backgrounds in biology and medicine, associated -omics disciplines, informatics, computer sciences, statistics, mathematics, and even social sciences, all of whom bring different epistemic frames reflecting their disciplinary training (Fagan 2017). Relatedly, others have shown how the interdisciplinary spaces of systems biology and systems medicine are characterized by different ideas about what the most significant aspects are that distinguish “systems” approaches from other biomedical approaches, and about where research efforts should be directed (see Green 2017).

Hence, the conceptual negotiations around “personalized,” “stratified,” and “precision” medicine on which this paper focuses are occurring against this background of biotechnological innovation. The “personalized,” “stratified,” and “precision” concepts are generally mobilized to describe the kinds of futures that data-intensive and predictive or systems approaches could or should enable. Indeed, formidable claims have been made about their potential to improve and re-direct medicine and healthcare. Particularly provocative and vocal examples have been claims made by Hood and colleagues (Flores *et al*. 2013; Hood and Friend 2011; Hood, Balling, and Auffray 2012) who have prophesized that we are at the brink of a paradigm shift; a medical revolution that will transform healthcare in the near future. Hood and colleagues predict that in the next few years, each patient will be surrounded by a virtual, longitudinal, multi-source and heterogeneous “data cloud” which can be interpreted to predict risks of disease before onset, enabling medicine to focus on optimizing health and wellness rather than on treating illness. This new generation of medicine will be personalized by accounting for individuals’ unique biological, environmental and lifestyle characteristics. Personalization will be accompanied with a participatory system where patients and citizens contribute their medical and health data to the benefit of research and are “empowered to take responsibility” over their own health by keeping track of their health data and modifying lifestyle in response to personalized preventative medical information.

Hood and colleagues’ vision of “predictive, preventive, personalized and participatory” medicine – what they call “P4 medicine” (Hood, Balling, and Auffray 2012) – is, however, only one example of a cluster of visions that have been articulated in the last two decades. Several terms have emerged that aim to capture, in a word, the new and transformed medicine that will soon be enabled, the most widely used of which have been “personalized” medicine, followed by “stratified” and “precision” medicine. For this reason, we focus on these three terms.

Existing theoretical and empirical contributions to the sociology of expectations have demonstrated how new and emerging biotechnologies and techno-scientific innovations are characterized by future-oriented expectations and visions that are manufactured and attached to the technologies as their “promise” (e.g. Broer and Pickersgill 2015; Borup *et al*. 2006; Brown and Michael, 2010; Brown, Rappert, and Webster 2000; Moirera and Palladino 2005). These expectations are “a way of attracting and synthesizing different kinds of capital” (Broer and Pickersgill 2015, 48), including economic, but also social, cultural and symbolic capital (Bourdieu 1986), that help to enroll different actors in support of particular research agendas and innovation models. The expectations and visions that articulate them play a performative role in relation to new technologies: the future promises that are manufactured by those visions that gain dominance in turn shape economic, social, cultural and symbolic forms of investment that are directed at the realization of particular kinds of futures (often at the expense of others) (Brown and Michael, 2010).

Moirera and Palladino (2005), among others, have argued that discourses around biomedical innovation tend to deploy a rhetoric of “hope” whereby research and scientific developments are justified by the promise of new treatments or “miraculous” medical innovations. These hopes and promises can, in turn, be carried by particular terms or labels via the connotations that they carry. Hedgecoe (2003), for example, has shown how the term “pharmacogenomics” was mobilized as a rhetorical device to gain support from policymakers and funders for research topics grouped under the term, by tapping into the “hype” surrounding the word “genomics.” Similarly, the terms “personalized,” “stratified,” and “precision” medicine have been mobilized by key political and biomedical actors in contextual ways that invoke particular associations and connotations. They have also been used in ways that are embedded within and support politically and economically motivated constructions of the “responsible citizen” subject, which draw from contextual articulations of the duties and rights of individuals in relation to collectives, including the state.

While the use and choice of the “personalized,” “stratified,” and “precision” terms is significantly directed, especially in policy but also biomedical discourses, by the expectations and promises of a better future, the choice and definition of the terms are also shaped, especially in biomedical discourses, by what Moirera and Palladino (2005) have called the deployment of “truth.” This entails “an investment in what is positively known, rather than what could be” (Moirera and Palladino 2005, 67). In practice, this implies a kind of “epistemic modesty” (Will 2010) – the deployment of what is “realistic” and what new biomedical technologies can actually deliver, in opposition to, or as a warning against, (what are framed as unrealistic) promises and hopes around “personalized,” “stratified,” and “precision” medicine. While both “hope” and “truth” function as rhetorical devices that are used to make future-oriented claims in order to shape the present, different expectations and articulations of possible futures are positioned differently in terms of the epistemic status and value they are claimed, by different actors, to carry in the marketplace of ideas.

We argue, then, that to understand the meaning and implications of “personalized,” “stratified,” and “precision” medicine, we must look, not into the future, but at *how* the future as a temporal abstraction is constructed, managed, invested in, by whom, and under what conditions (Brown, Rappert, and Webster 2000).

## Personalized medicine

**“**Personalized medicine” has been the most common label applied in biomedical discourses to describe the future potential and aims of translational big data, predictive and systems biomedicine (Pokorska-Bocci *et al*. 2014). It has also become the dominant term used in European policy discourses, having been adopted by the European Commission as the preferred term to denote related emerging technologies and research in the context of European healthcare systems. There are, however, two key sources of dispute around the meaning and scope of the concept: first, whether or not “personalized medicine” is an appropriate term to use; and second, what exactly the concept does or should encompass (De Grandis and Halgunset 2016). Both disputes are related to the future-oriented ways in which the concept is applied, and to the contextual ways in which its meaning is interpreted and delineated by different policy and biomedical actors.

In the European policy context, EU funding programs have been investing in “personalized medicine” since the 7th European research and technological development framework program began in 2007. The current investment includes the Horizon 2020 program and its Innovative Medicines Initiative, aimed at accelerating the development of effective preventative and diagnostic tools. Through these programs alone, over 3 billion Euros of EU funding has been invested to support research grouped under the label (Nimmesgern, Benediktsson, and Norstedt 2017). In 2015, the European Commission coined a definition of personalized medicine according to which

personalised medicine refers to a medical model using characterisation of individuals’ phenotypes and genotypes (e.g. molecular profiling, medical imaging, lifestyle data) for tailoring the right therapeutic strategy for the right person at the right time, and/or to determine the predisposition to disease and/or to deliver timely and targeted prevention. Personalised medicine relates to the broader concept of patient-centred care, which takes into account that, in general, healthcare systems need to better respond to patient needs (Council of the European Union 2015).

This definition was applied by the Horizon 2020 program, which identified personalized medicine as a core theme, and highlighted better understanding of disease etiology at a systems level as a key research priority (European Commission 2017). The Horizon advisory group justified the choice of “personalized medicine” over alternative terms by noting that this “term best reflects the ultimate goal of effectively tailoring treatment based on an individual’s ‘personal profile’, as determined by the individual’s genotype and phenotype data” (European Commission 2016a, 19).

The European Commission’s and associated organizations’ terminological and definitional choice is explicitly connected with the aim and hope that the translation of systems approaches to healthcare will enable both a preventative model of medicine and, relatedly, the incorporation of greater patient participation and accountability to the management of their own health. Illustrative is the Strategic Research and Innovation Agenda (SRIA) developed by the EU-funded PerMed project, and aimed at shaping Europe’s vision for personalized medicine. The SRIA called for “a paradigm shift for all citizens, researchers and national healthcare systems,” underlining the importance of patient participation and responsibility in the ownership and control of personal health data (PerMed2020 2015, 2). Similarly, the European Commission’s 2015 *Making Access to Personalized Medicine a Reality for Patients* conference, organized to address the integration of personalized medicine into EU healthcare systems, emphasized the need to develop a patient-centered approach for “the benefit of all Europe’s patients, now and in the future” (Luxembourg Ministry of Health and European Commission 2015). The International Consortium for Personalized Medicine (IC PerMed) was initiated in 2016 to advance these aims, including 20 public and private non-profit health research funding and policymaking organizations (European Commission 2016b).

The choice of the term “personalized medicine,” together with its associated emphasis on patient-centered approaches, citizens’ responsibility and control over their health through predictive healthcare models, brings to the fore the future aspirations driving research investment in the European policy context. The investment is shaped by hopes for tailored treatments in terms of providing “the right therapeutic strategy for the right person at the right time,” intertwined with the conceptualization of personalized medicine as a “paradigm shift” not only for researchers but also for healthcare systems and citizens. The aim of placing citizens “at the center of healthcare” incorporates a desire to enable not only sharing and ownership but also responsibility towards one’s data and one’s health. This “responsibilization” in relation to health implies “personalization” of healthcare “in the sense that more responsibility for management of healthcare is primarily laid on or taken by individuals or their carers rather than medical professionals” (Nuffield Council of Bioethics 2010, 30).

In contrast to European policy discourses, in the biomedical literature, although personalized medicine has been celebrated as the enabler of “individualizing patient care across the continuum (from health to disease) … resulting in an unprecedented customization of patient care” (Ginsburg and Willard 2009, 278–279), the term has a wide scope and inconsistent application and has also incited derision. Indeed, some have argued that the idea of “personalization,” at least as treatment individualization, is in practice demonstrably fallacious (Nicholls *et al*. 2014). There have been several attempts to clarify the meaning and scope of the concept. Perhaps the most influential of these has been a definition proposed by Schleidgen and colleagues (2013) based on a systematic literature review of biomedical journal articles. We will discuss this definition in some depth, because both the definition itself and the methods through which it was derived illustrate how the concept has been delineated and negotiated in biomedical literature more generally.

Schleidgen and colleagues argue that the vagueness of “personalized medicine” has unduly complicated public discourses around the concept, and fear that some stakeholders might use it to further their particular (especially economic) agendas in morally unacceptable ways. They contend that a shared understanding of the term is required to mitigate these problems, but also that adequate analytic criteria should be used to construct the definition to avoid arbitrariness. Consequently, they propose and apply several *a priori* analytic criteria against which definitions can be evaluated. Key amongst these is that an adequate definition of personalized medicine cannot diverge from what medical technologies and knowledge can actually deliver. They argue that definitions including such goals as “improving tailoring of prevention and therapy” at an individual level are false, due to the impossibility of realizing this goal in practice. Their approach exemplifies the deployment of “truth” (Moirera and Palladino 2005) as a strategic (future-making) device in opposition to (what are framed as) too “hopeful” or unrealistic promises that circulate in policy and political discourses, where the (especially economic) agendas advanced are taken to be ethically partial.

Indeed, Schleidgen and colleagues state unequivocally that personalized medicine “is not medicine with a special focus on the interests and preferences of the individual patient” and it “is not related to the term patient-centered medicine,” nor can patient-centered medicine be realized solely through personalized medicine (11). That some (including the European Commission) have conflated personalized and patient-centered medicine demonstrates, for Schleidgen and colleagues, that stakeholders can misuse the conceptual ambiguity of the term by inflating it with optimistic meanings according to their own agendas when, *in reality*, “tailoring means no more than stratification” (10). Several biomedical commentators have agreed: Abettan (2016, 426), for example, argues that “personalized” medicine “is catchy but misleading” for similar reasons, while Honoré (2012, 497) has remarked that given “all the complex processes that drugs undergo in the body, determining the pharmacokinetics and pharmacodynamics in detail will never be possible” and related new methods can thus only be described as stratified, not personalized dosing.

The definition proposed by Schleidgen and colleagues (2013, 10) is that personalized medicine “seeks to improve stratification and timing of health care by utilizing biological information and biomarkers on the level of molecular disease pathways, genetics, proteomics as well as metabolomics.” Yet, rather than serving as a resolution to personalized medicine’s conceptual ambiguity, this definition and the criteria based on which it was constructed can themselves be taken to exemplify context-dependent interests and aspirations that have become attached to “personalized medicine” in biomedical contexts. Despite their condemnation of the possible co-optation of the concept by stakeholder interests, Schleidgen and colleagues themselves arrive at their definition building on biomedical literature alone. They promote their own definition as adequate for public engagement and communication, even though they acknowledge the widespread use of the concept in public as well as academic discourses. This seems either to rely on the assumption that biomedical literature is unbiased rather than itself representing the views of particular stakeholders (De Grandis and Halgunset 2016), or to be an attempt to assert authority over the definition of “personalized medicine” (via deploying “truth,” with the corresponding claim to know what the truth is). In the latter case, biomedical scientists are framed as the stakeholders with the most significant “expertise” or authority to define what “personalized medicine” means and can mean, *realistically*. Yet, rather than protecting “personalized medicine” from stakeholder bias, Schleidgen and colleagues effectively appoint one stakeholder group as the (only) legitimate delineator of what “personalized” medicine can be, to the extent that other stakeholders’ conflicting interests, including patient-centeredness, are classed as “misuse” of the concept. Indeed, Schleidgen and colleagues’ definition can be seen as privileging the biological and molecular interpretation of “personalization” as group-level treatment stratification, to the explicit and purposeful exclusion of patient-centered interpretations.

Simmons and colleagues (2012) have noted that personalized medicine often comes to serve as a catch-all term used synonymously with genomic medicine, with this equation having the effect of narrowly focusing attention on the application of genomic and related technologies, while diverting attention from broader applications of personalization in health and biomedicine. This includes marginalizing environmental and socioeconomic triggers of health and disease as aspects of personalization. By contrast, some scholars especially in the humanities and social sciences have suggested that this narrow interpretation should be seen only as one part of a broader notion of personalization. Cherny and colleagues (2014), for example, have proposed the term “biologically personalized therapeutics” to denote these aspects, arguing that biological aspects should not be equated with, but seen as a part of, a more holistic conception of “personalized medicine” that would focus on patient-centered care and view patients as “whole persons.”

Relatedly, Schleidgen and colleagues’ emphasis on what medicine can in practice deliver and their desire to disentangle personalized medicine from patient-centered care not only render the European Commission’s aspirations for personalized medicine as misuse of the concept, but also side-line the future-oriented motivations that foreground these aspirations. By excluding patient-centered care and more inclusive interpretations of personalization as unrelated to personalized medicine, their definition tends to foreclose possible futures where the boundaries of both “personalization” and “medicine” more comprehensively incorporate data from “systems” beyond the traditional focus of medicine; namely, the environmental and/or socio-economic. These broader notions of personalization and medicine are in part what shape related research investment in the European policy context, illustrating a disconnect between policy aspirations, and agendas advanced by Schleidgen and colleagues’ and other biomedical commentators. The disconnect derives from differently articulated priorities linked with different stakeholders’ agendas and values.

It is also noteworthy that while existing research on patients’ and publics’ perceptions of personalized medicine is limited, the research that does exist (Budin-Ljøsne and Harris 2016; Gray *et al*. 2012; Heusser 2015; McFarland 2014) suggests that patients and publics generally place little emphasis on biology-based treatment individualization or genomics for “personalized medicine.” Rather, they highlight aspects including patient empowerment, ability to participate in medical decisions, better physician-patient relationships and being in contact with one’s physician as key to personalization, thus understanding patient-centered care as central to personalized medicine. In Heusser’s (2015, 78) words, their conceptions “are more humanistic in nature:” personalized medicine for the “lay public does not match the biological meaning attributed to the term” but “is automatically associated with a more holistic concept of the ‘person.’” Patients as key stakeholders are actively involved in negotiating and delineating the meaning of personalization in the context of their own lives, in ways connected with responsibilization and increased individual accountability over health management.

While “personalized medicine” has been the most prominent term in biomedical literature as well as being adopted by the European Commission among others, some have argued that the term “stratified” should be used instead of “personalized” to evade the kinds of “overly optimistic” associations relating to the “personalization” concept that Schleidgen and colleagues wished to avoid.

## Stratified medicine

Some policy influencers and academic biomedical commentators have advocated “stratified medicine” as a preferable term to “personalized medicine,” in ways that mirror critiques of the latter expressed in biomedical literature. The World Health Organization (WHO), for example, has argued that “stratification” more accurately “reflects the realistic effects of medicines at population level, while the term ‘personalized medicine’ reflects the possibly overambitious promise of individualized unique drug targeting and development” (2013a, 180). Similarly, the UK Academy of Medical Sciences (AMS) adopted the concept of “stratification” instead of “personalization,” defining it as “the grouping of patients based on risk of disease or response to therapy by using diagnostic tests or techniques” (2013, 5).

Thus, while stratified medicine has also been used to denote visions of the future of healthcare promised by data-driven predictive or systems biomedicine, these visions have simultaneously been contrasted against the “misleading and segmented” nature of “personalization,” compared to which “stratification” has been taken as a concept that “better captures the hopes and aspirations of this new branch of medicine” (Chataway 2012, 732). “Stratification” can thus be seen as more “epistemically modest” and “truthful” conceptualization than the “hopeful” and promissory framings of “personalization.” Yet, like “personalization,” what exactly the “stratification” concept implies is contested, and how “stratified medicine” differs from “personalized medicine” is subject to disagreement. Some biomedical commentators have drawn distinctions between the two as different approaches, whereby stratified medicine amounts to “the definition of population subgroups on the basis of estimates of their differences in disease susceptibility and prognosis, or responses to treatment” while personalized medicine has been “used to describe the explicit focus on individual patients” (Matthews *et al*. 2014, 16). Others, however, have argued that stratified medicine is a “prelude” to personalized medicine (Maglo 2012, 138), including the AMS (2013b) which (while currently preferring the “stratification” concept) argued that stratified medicine is “an approach to therapy that forms a key step on the path towards personalized healthcare.” They thus mirror the European Commission and other advocates of “personalized medicine” in understanding personalization as the ultimate end goal to be pursued.

A 2015 joint forum meeting co-organized by the UK AMS, however, concluded that the language of stratification was potentially problematic due to its exiting associations with social divisions – especially stratification along ethnic and socioeconomic lines – which could render the concept misleading or problematic when applied in public communication or engagement (AMS 2015). Indeed, based on research with UK stakeholders, Innovate UK and Sciencewise reported that lay representatives’ initial responses to the “stratified medicine” terminology were negative (Farrow, Swinn, and Bua 2014). This was because, firstly, the term was seen as inaccessible and, secondly, it evoked associations between genetic stratification and social stratification along racial and other sociodemographic lines, and incited concerns over the implications of this. Young people in particular associated the idea of stratification into treatment groups with racial profiling. Innovate UK and Sciencewise thus concluded that when it comes to public communication, “stratified medicine might not be the right term for this task” (Farrow, Swinn, and Bua 2014, 24). While they noted that members of the public had suggested alternative terms including “personalized medicine,” such alternatives were also criticized as potentially misleading in ways resembling broader critiques of the “personalization” term, especially in relation to associations of treatment approached being based on individual rather than group characteristics. Communication about medicine, they argued (in ways that recall the deployment of “truth” in contrast to “hope” by Schleidgen and colleagues), should not raise hopes that cannot be realized (Farrow, Swinn, and Bua 2014).

Similarly, based on interviews and ethnographic research with stakeholders in the US, Juengst and colleagues (2016) argue that the term “stratified” has failed to gain traction in the US context due to its associations with racial and income stratification, genetic and healthcare discrimination, and social injustice. To avoid these associations and related social and political concerns around the language of “population stratification,” Juengst and colleagues argue that a term with more neutral, if not positive, connotations was needed. The term that has become hegemonic in the US is “precision medicine.”

## Precision medicine

In the context of US policy and biomedical discourses, Juengst and colleagues (2016) argue that a wave of “rebranding” or rhetorical reform has taken place, with opinion leaders abandoning the previously more widely used “personalized medicine” term in favor of “precision medicine.” In their 2011 *Towards Precision Medicine* report, the National Research Council (NRC) of the US National Academies encouraged “precision medicine” research, describing it as having the “ultimate endpoint” of enabling “the selection of a subset of patients, with a common biological basis of disease, who are most likely to benefit from a drug or other treatment” (NRC 2011, 37). As a justification for their choice of term, the NRC (2011, 12) noted that in comparison with “personalized medicine,” “precision medicine” is “less likely to be misinterpreted as meaning that each patient will be treated differently from every other patient.” Charles Sawyers, who co-chaired the NCR committee that produced the *Towards Precision Medicine* report, commented that while the committee “spent a considerable amount of time on whether we were talking about personalized medicine or precision medicine,” the choice of “precision” was motivated by their wish to “convey a more precise classification of disease into subgroups that in the past have been lumped together because there wasn’t a clear way to discriminate between them” (cited in Katsnelson 2013, 249).

The NRC’s promotion of “precision medicine” gave the term some purchase over alternatives (especially) in the US context. In January 2015, US president Barak Obama launched what came to be called the Precision Medicine Initiative (PMI), investing $215 million to realize the initiative’s mission of enabling “a new era of medicine through research, technology, and policies that empower patients, researchers, and providers to work together toward development of individualized care” (White House 2015a). While the NRC initially used the “precision” term to indicate subgroup classification, under the PMI, precision medicine is given a notably different definition, as “an approach to disease treatment and prevention that seeks to maximize effectiveness by taking into account individual variability in genes, environment and lifestyle,” through a “combined analysis of biological, environmental, and behavioral factors that contribute to health and disease” (NIH 2015, 6). It is promoted as a “bold new research effort to revolutionize how we improve health and treat disease” (NIH 2018). Mirroring the European Commission, the FDA, which was awarded $10 million for the development of new regulatory and evaluation approaches for the PMI, noted that the goal of precision medicine “is to target the right treatments to the right patients at the right time” (FDA 2017).

At the center of the PMI is the so-called “All of Us” research program administered by the National Institute of Health (NIH) with the aim of recruiting one million volunteers to form a longitudinal cohort reflecting the demographic diversity of the US population. They hope to build a databank large enough to facilitate detection of “associations between genetic and environmental exposures and a wide variety of health outcomes” to enable “the exploration of biological, clinical, social, and environmental determinants of health and disease” (NIH 2015, 6–7). The objective is to collect and record biospecimens, physical measurements, electronic health records, participant-provided data collected through surveys, and even mobile and digital health data from health, fitness and wellness devices and applications. The databank has a wide range of anticipated applications including quantitative estimates of disease risk, optimization of screening and prevention strategies, and is intended to be widely accessible including to “citizen scientists” and “engaged participants” (NIH 2015, 19–20). This reflected the Obama administration’s stated goal of ensuring that citizens and/or customers have access to their own health data so that “in addition to treating disease, we can empower individuals and families to invest in and manage their health” (White House 2015b). In Obama’s words, precision medicine “helps us create a genuine health care system as opposed to just a disease care system,” allowing “each of us to have sufficient information about our particular quirks … that we can make better life decisions” (White House 2016).

Notably, there is significant overlap between the definitions and conceptualizations of “personalized medicine” applied in the European policy context, and the definitions and conceptualizations of “precision medicine” applied within the PMI. Indeed, in launching the PMI, Obama seems to have taken the two terms quite synonymously: while opting for precision medicine, he noted that “in some cases people call it personalized medicine” (White House 2015c). The PMI conceptualization of precision medicine significantly departs from that initially promoted by the NCR. Its similarity to European policy discourses around personalized medicine, however, highlights how different terms carrying different associations have been chosen and used in different policy contexts, but promoted in similar ways to describe the promise of big health data and predictive systems level analysis.

The choice of “precision medicine” in the US policy context reflects both critiques and undesirable connotations of alternative terms, and how they manifest in the sociohistorical and socioeconomic context of the US. The “precision” term avoids critiques of the term “personalized” by evading the latter’s hopeful but possibly overambitious promise, while still enabling policymakers to apply the rhetorical force of medicine “tailored to individuals’ lifestyles, genes, environment and preferences.” It avoids associations with social divisions aroused by “stratified medicine,” while simultaneously reframing the group stratification and medical profiling that predictive and systems approaches enact as ethically neutral or even positive “precision.” The latter rhetorical quality is particularly noteworthy in the context of American politics of race, with a long history of both racial segregation built on scientific racism, and prominent and powerful anti-racist politics and opposition in response to this segregation. Applied as an umbrella term, it also enables the joining of genomics (and other -omics fields) with other ethically complex and controversial research modes such as biobanking and data mining of electronic health records under the language of “precision.” It has associations with notions such as “precision equipment,” and even resonates with “military precision” and the appeal of phrases like “precision bombing” in contemporary American culture (Juengst *et al*. 2016). However, like the European Commission’s emphasis on citizens’ responsibility towards personal health data, the PMI is intertwined with the aspiration of enabling individuals to invest in and manage their own health – to “make better life decisions.” The initiative, like the European Commission’s investment in “personalized medicine,” is connected with health “responsibilization,” which brings with it a tension between citizens’ empowerment rhetoric in relation to health and increased individual accountability over health.

While “precision medicine” has not been as popular as “personalized medicine” in biomedical literature (Pokorska-Bocci *et al*. 2014), the concept has likewise been defined and applied by biomedical researchers and scholars across disciplines in ways closely resembling the language used to define personalized medicine. This includes definitions of precision medicine as “treatments targeted to the needs of individual patients” (Jameson and Longo 2015, 2229) and healthcare “individually tailored on the basis of a person’s genes, lifestyle and environment” (Hodson 2016, S49). Some have described precision medicine as “patient-centered and multifaceted extension of personalized medicine” (Matthews *et al*. 2014, 16). The WHO (2013b, 7–8) on the other hand stated that whereas “the term ‘stratified medicine’ reflects the realistic effect [of using myriad data to define individual disease patterns] on patient/population-level, ‘precision medicine’ reflects the clinical consequences — a better treatment.” In biomedical as well as policy discourses, the respective meanings attributed to personalized and precision medicine are thus also muddled in important ways.

## Future promises, present values

The futures that are imagined for data-intensive, predictive and systems biomedicine are multiple and embedded within broader contextual frames, but there are also overlapping themes that cross from one future vision to another, yet manifest somewhat differently in ways that reflect contextual variances and different agendas. “Personalized,” “stratified,” or “precision” medicines are emergent phenomena, where the process of their emergence is one of delineation, negotiation and contestation in relation to the activities that they encompass and the roles they (should) play in broader contexts of healthcare and biomedicine. The aims that are stated, and those that are implicit, in each version of personalized, stratified or precision medicine that are articulated in policy and biomedical discourses reflect the interests and aspirations of the articulators, which relate not only to what the future should look like, but also to what matters in the present.

In the policy context, shared aspirations to increase individuals’ capability to take control of their own health are centrally embedded in political discourses in both the US and Europe. Such control is promoted as empowering and desirable, highlighting patients’ and citizens’ agency and the value of increased health knowledge and capacity. However, these discourses also carry connotations of “responsibilization” and its possible implications.^1^ Increasing individuals’ control over their own health also involves increasing individuals’ responsibility for their health, which in turn easily translates into heightened expectations and even obligations around maintaining and promoting personal health. Whereas many (especially biomedical commentators) have celebrated the revolutionary and transformative potential of predictive, big data, and systems approaches in this regard, some bioethicists and scholars in the social sciences have expressed concern over the possible implications of responsibilization as a form of increased surveillance and medicalization. This carries implications for what the role of the individual citizens in systems medicine is or should be, as citizens are (re)positioned as agentic and increasingly accountable actors in health management and diseases prevention.

These concerns recall Beck’s (1992) argument that we are living in a “risk society” where omnipresent risks, ranging from environmental risks to terrorism to health risks, dominate public discourses in unprecedented ways, positioning ignorance and lack of knowledge about these risks as a dangerous failure to anticipate and prepare for them. Disaster arises from not-knowing while knowing enables mitigation and control, which in the context of healthcare is being framed as accountability over risks of illness and disease mitigated through individual agency. Vogt, Hofmann, and Getz (2016), for example, have argued that the aims of these new and emerging approaches to biomedicine, which include as a central characteristic a focus on prediction and prevention, have the implication of rendering health and illness actionable as well as controllable. As noted above, biomedical commentators such as Hood and colleagues have seen actionable health information as empowering because it enables individuals to track and modify their lifestyle and health behaviors to prevent illness. Vogt, Hofmann, and Getz, however, worry that the impact will be the medicalization of “health and life itself” achieved through medical(ized) social- and self-surveillance. This also evokes Foucault’s (1991) “panopticism” thesis: “biomedicine would strengthen its grip on what it means to lead a healthy life, and even lifestyle, living itself, would be grounded in a continuous, technologically based monitoring of risk-factors” (Vogt, Hofmann, and Getz 2016, 320). This, in turn, can “lead to a damaging labelling of aspects of life *as medical* and displace other valid goals, values and ways of understanding and tackling life” (320). Thus, “at stake is no less than a person’s own ability to state ‘*I am well*’ without having to consult a computational mirror image” (320, original emphasis). Indeed, some have taken this further to advance dystopia-like prophecies centered on medical surveillance and control penetrating into our every-day lives: the post-genomic era is “setting the stage for a digital panopticon” which will result in “an emptying or obliteration of life (dissolving individuality into pure data)” (Zwarts 2016, 69, 85).

The drive towards responsibilization has, however, contextual manifestations in the present as well the envisioned ‘panopticized’ future. These are influenced by broader economic and political drivers around healthcare investment, but structured by key differences between the US and European healthcare frameworks. Both contexts have been shaped by economic downturn and reduced public spending at the wake of the 2008 global financial crisis, which placed extra pressure on existing demands on governments internationally to produce efficiency savings including from health resources (Rooshenas *et al*. 2015). The drive towards citizen responsibilization and investment in the hope of predictive and preventative (personalized or precision) medicine are aligned with political motives to cut public spending on healthcare generally, but the politics of individual responsibility over health are particularly resonant in the context of American individual-centered culture.

Healthcare in the US is already, and has long been, fully marketized as a private expense, structured by an employment-based health insurance system. In European countries, however, the public sector has largely been expected to bear the costs of healthcare, and principles of entitlement to healthcare access for all (through public/national healthcare systems like the UK National Health Service) are strongly embedded in the cultural value system. European welfare model-based public healthcare systems have been grounded on the assumption that risk of unforeseen illness is approximately equally shared and should therefore be pooled (Busby and Martin 2006). However, the translation of predictive and preventative personalized, stratified or precision medicine frameworks for healthcare poses a challenge to assumptions of equity and shared risk and, arguably, carry more disruptive implications for European systems than for the marketized US system. Due to the differences between their underlying ethical principles, the normative implications for existing healthcare systems will vary in relation to ideas around how and why the healthcare system itself should be provisioned and what the role of individual citizens should be in relation to the state.

Yet, within policy discourses, both “precision” and “personalized” medicine initiatives are also entangled with promises of a new model of citizens’ and publics’ participation, envisioned as (inter)active and collaborative with healthcare and research professionals, policymakers and regulatory bodies. These discourses frame healthcare around a social contract model of benefits and obligations where taking responsibility over one’s health is enticed in exchange for personalized or precision (or stratified) healthcare (Davies *et al*. 2017). The connection between citizenship and biomedical science in general and genomics in particular has been analyzed by others (e.g. Busby and Martin 2006; Rose and Novas 2005; Sabatello and Appelbaum 2017), especially in relation to the human genome project and emergence of national and group identities based on population genomics (e.g. Benjamin 2009; Schwartz-Marin and Restrepo 2013; Schwartz-Marin and Silva-Zolezzi 2010). In the context of the PMI, discourses around “precision” healthcare are intertwined with citizenship discourses promoting shared national interests and values by appealing, not only to the liberal individualistic ethos embedded in the American value system, but also to a shared national identity – “All of Us,” as the core research program is labeled (see also Sabatello and Appelbaum 2017). Although less influential, discourses around a shared (cross-)European identity and related citizenship rhetoric are also advanced by the EU in ways that embed the European Commission’s promotion of personalized medicine. Because a collective identity or sense of belonging have been taken as necessary for effective shared governance, a rhetoric of “European” identity and “European citizenship” is embedded in the EU framework (Kantner 2006) which also shapes how personal responsibility over health is interpreted in the context of “European” healthcare for “citizens of the European Union.”

In sum, then, for policymakers both in Europe and the US, emphasis is placed on increasing the effectiveness of healthcare through prevention, responsibilization and, consequently, a diminished burden of care carried by state and public health authorities. The rhetoric mobilized – emphasis on “personalization” and patient-centered care by the European Commission versus individualization and “precision” in the US – reflects the need for public approval of policy initiatives and investment. Language applied in policy discourses must reflect both policymakers’ hopes for what the policies can and should deliver, but also harness national and regional public discourses and citizens’ hopes around what “better” or improved healthcare could or should mean.

While the most persistent thematic around personalized, stratified, and precision medicine is the future-oriented nature of related discourses not only in policy but also biomedical contexts, biomedical commentators have been more invested in articulating, in Moirera and Palladino’s (2005, 67) words, what is “positively known” about the translational potential of new biomedical research modes. As noted above, this deployment of “truth” in contrast (or at least in addition) to “hope” presumes that those who articulate it know what the “truth” is, and it also implies a kind of epistemic modesty, which is positioned differently in terms of the epistemic status it is implied to carry. This is most strongly illustrated by the rationales advanced by biomedical commentators for the use of the “stratification” rather than “personalization” term as a more realistic concept that accounts for what the new biotechnologies can actually or “truly” deliver. It is also illustrated by the definition of “personalized medicine” advanced by Schleidgen and colleagues: by prioritizing biomedical scientists’ conceptualizations of “personalization,” they position biomedical discourse as either untainted by the kinds of economic biases that shape policy discourses, or, at least, as having more authority (derived from “expertise”) to know and define what “personalized medicine” can “really” mean.

Biomedical and academic articulations of “personalized,” “stratified,” and “precision” medicine also need to be contextualized, however, in relation to the broader landscape of academic and clinical research where scientists compete for research funding in ways constrained by the conditions of limited research and healthcare investment. Proponents of “personalized,” “stratified,” and “precision” medicines, both in Europe and the US, compete not only against each other but also against the claims and promises made by other research areas in the marketplace of ideas to secure investment in their projects. Biomedical scientists’ efforts to define and delineate “personalized,” “stratified,” and “precision” medicines can be seen as efforts to construct a marketable identity (and agenda) for an emerging biomedical research area, and as efforts to distinguish their epistemological and ontological approach to this area from other competing approaches. They can simultaneously be seen as efforts to delineate the “newness” of their approach from past research modes to secure public and policy interest and investment. Choosing and defining a term that is then claimed as *the* label that collates different new big data, predictive, and systems approaches is a way of unifying a cluster of emerging biomedical research practices and activities in an intelligible way (Powell *et al*. 2007). This unification, achieved via the demarcation of an ontological and epistemological territory through terminological and definitional work, simultaneously foregrounds and is reinterpreted in policy contexts, positioned within national and regional political and policy agendas, and (re)framed by and for patients and publics in relation to their interests and needs.

The open and fluctuating boundaries of “personalized,” “stratified,” and “precision” medicine reflect, then, the context-dependent nature of biomedical and techno-scientific innovation, and how they come and are made to matter for and by different actors and groups in different ways. The chosen terms and their definitions, as labels and boundary delineations, mirror different interests, agendas, and visions around big data integration, preventative and systems approaches to biomedicine, in ways that make the terms and definitions work as marketing tools especially around research investment (Powell *et al*. 2007). Which term gains dominance in which context is, on the other hand, likely to shape biomedical innovation in the present in contextual ways: if we envision and invest in the “ultimate goal” of “personalized” medicine as treatment individualization embedded within a patient-centered healthcare model, for example, the direction of medical and healthcare innovation will differ from one in which the future of biomedicine was envisioned to be treatment stratification based on biomarkers and genetics. These delineations are also connected with and shape how patients and citizens are positioned (and position themselves) in relation to related discourses and what kinds of roles they are envisioned (and envision themselves) to have. Different actors have different ideas about what their position is, or should be, in relation to biomedical innovation and its future; choices over preferred terms also reflect attempts to negotiate this position at a personal or individual level.

The ongoing negotiations and contestations over the present meaning and future implications of personalized, stratified, and precision medicine derive from uncertainties around what new and emerging big data, predictive and systems biomedicine actually imply in practice, and what they can and should become. These open questions reflect how new biomedical technologies, innovations and approaches are socially navigated, contested, and delineated in the context of their emergence.

## Conclusion

In dispute when it comes to which term should be chosen to denote the potential of new and emerging big data, predictive, and systems approaches to biomedicine are the social ideals and values that the different terms carry as their associations: which terminology and definitions gain dominance, where, and why is a result of struggle over these ideals, values, and priorities. The terminological debates, then, and the related epistemic and ontological tensions around where focus is placed and how conceptual boundaries are drawn are a matter of struggle over what a desirable and realizable future does and does not, and should and should not look like. Each term taps into different interests and links to future imaginaries that can then be used to gather support for and investment in emerging research activities. The future visions that prevail or gain prominence in different contexts ultimately reflect the power of, and investment in, the future visions and promise that has been manufactured.

Our aim has been to map how the contextual conceptual, terminological and definitional boundary work and the future-oriented temporality that characterizes it are a key aspect of how biomedical innovations are made meaningful. The diversity of concepts that invoke “hopes” and “truths” about various biomedical and healthcare futures that could or should materialize also shape whose interests will ultimately be served by biomedical innovation. How and by whom these futures are manufactured matters because the temporal and contextual nature of the discourses around personalized, stratified, and precision medicine are central to how they are conceptualized and understood.

Existing analyses around the potential implications of big data, predictive, and systems focus in biomedicine – such as analyses focused on responsibilization, increased medicalization and surveillance that they can give rise to – are necessary and valuable, as are analyses focused on delineating the possible meanings and scope of “personalization,” “stratification,” and “precision.” However, rather than pinpointing which of these terms is the “correct” one or delineating the “true” meaning of each, to know how we should critically approach the concepts we need an awareness of the discursive contexts in which they are mobilized. This is because the context ultimately structures the social and ethical implications that “personalization,” “stratification,” or “precision” will have for medicine and healthcare systems, and for different stakeholders. As big health data, predictive and systems-level analysis are, themselves, emergent phenomena, the terminology applied in the discursive spaces around these new biotechnologies and approaches cannot be abstracted from their context. Rather, when we apply the “personalization,” “stratification,” and “precision” terms, we invoke particular associations, connotations, “hopes” and “truths” that are part of pre-existing epistemologically and ethically loaded discourses that reflect broader and weightier struggles over what is a good future.

## Note

1 See Nuffield Council of Bioethics (2010) for in-depth discussion of responsibilization.
